# Knowledge, attitudes, and practices regarding childhood epilepsy among parents of children with epilepsy: a questionnaire-based study

**DOI:** 10.1038/s41598-026-50907-2

**Published:** 2026-05-18

**Authors:** Shou-Yun Ren, Li Qiao, Juan Li, Jun-Ji Hu, Chun-Yan Fan

**Affiliations:** 1 Department of Pediatrics, Zibo First Hospital, Zibo, 255000 Shandong China; 2 Department of Neurology, Zibo Changguo Hospital, Zibo, 255000 China

**Keywords:** Epilepsy, Parents, Knowledge, Attitude, Practice, Surveys and questionnaires, Health education, Diseases, Health care, Medical research, Neurology, Neuroscience

## Abstract

This study examined the knowledge, attitudes, and practices (KAP) among caregivers of children with epilepsy. A cross-sectional survey was conducted from September 2024 to February 2025 in two hospitals in Zibo, China. Structural equation modeling (SEM) was applied to explore the relationships among the KAP components. A total of 385 caregivers participated; most were female (59.5%) and urban residents (44.7%). Regarding education, 39.5% held an associate degree or higher, and 49.1% were employed. Most children were aged 7–12 years (45.7%). Mean scores for knowledge, attitudes, and practices were 19.76 ± 2.68, 23.43 ± 3.18, and 23.57 ± 2.52, respectively. Although overall knowledge levels met the threshold for adequacy, important gaps remained, such as uncertainty regarding vaccination safety. Attitudes were often negative, with 70.9% of caregivers believing epilepsy would adversely affect career and marriage prospects and 35.6% expecting social prejudice. In addition, some caregivers reported potentially unsafe practices, such as inserting objects into the mouth during seizures. SEM results indicated that knowledge was positively associated with attitude (β = 0.303, *P* < 0.001) and practice (β = 0.204, *P* = 0.001), while attitude showed a strong association with practice (β = 0.708, *P* < 0.001). Knowledge also demonstrated an indirect association with practice through attitude (β = 0.215, *P* < 0.001), although the magnitude of this effect was modest. In conclusion, caregivers of children with epilepsy demonstrated knowledge levels that met the adequacy threshold but exhibited persistent misconceptions, negative attitudes, and suboptimal caregiving practices. These findings suggest that improving caregiving behavior may require multifaceted interventions that address not only knowledge but also attitudinal and contextual factors.

**Supplementary Information:**

The online version contains supplementary material available at 10.1038/s41598-026-50907-2.

## Introduction

Epilepsy represents one of the most prevalent serious neurological disorders globally, affecting over 70 million individuals and characterized by recurrent, unprovoked seizures resulting from abnormal neuronal activity within the brain^1^. In pediatric populations, the global incidence is estimated at approximately 50–100 per 100,000 children annually, with prevalence rates ranging from 4 to 10 per 1,000 children across most populations^2,3^. This burden becomes disproportionately pronounced in low-resource settings, particularly within rural areas of developing countries, where both incidence and prevalence reach substantially elevated levels^4^. Despite the apparent predominance of focal over generalized seizures, only approximately one-third of pediatric cases can be definitively classified into established epilepsy syndromes^5^.

The unpredictable and chronic nature of epilepsy presents considerable challenges for affected children and their families, particularly regarding sustained care management and social adaptation. Children with epilepsy remain heavily dependent on familial support for daily disease management, with parents functioning as primary caregivers throughout the treatment trajectory^6^. Parental responsibilities encompass medication administration, seizure monitoring, emotional support provision, and coordination of follow-up care—all critical components for minimizing seizure recurrence, preventing injury, and promoting the child’s long-term well-being^7^. Previous research has demonstrated that elevated levels of parental knowledge coupled with supportive attitudes correlate significantly with enhanced treatment adherence and superior seizure control^8,9^. Consequently, strengthening parents’ capacity to recognize, comprehend, and manage epilepsy emerges as fundamental to effective home-based care and improved quality of life for affected children.

The Knowledge–Attitude–Practice (KAP) framework has gained widespread acceptance in health research as a tool for evaluating the cognitive and behavioral preparedness of target populations^10^. This theoretical model posits that enhanced knowledge facilitates positive attitude formation, which subsequently promotes beneficial health practices^11^. KAP surveys are frequently employed to identify knowledge deficits, assess health beliefs, and evaluate behavioral responses among caregivers, thereby providing valuable insights for designing health education interventions and service delivery systems^12^. Within the epilepsy context, KAP studies have proven instrumental in revealing caregiver misconceptions, emotional responses, and preparedness levels for seizure management and long-term care provision^13^.

In China, research examining the KAP status among parents of children with epilepsy remains notably limited. Existing investigations predominantly focus on isolated components—such as seizure-related anxiety or emergency response protocols—while failing to provide comprehensive assessments of caregiver knowledge, attitudes, and practices^14^. A recent multicenter survey involving 645 caregivers revealed that over 45% demonstrated inadequate knowledge and seizure management competencies, with rural families showing particularly pronounced deficiencies^15^. Furthermore, few studies have systematically explored the demographic and clinical determinants that influence parental KAP or examined the interconnections among these three domains.

To address this research gap, the present study aims to comprehensively evaluate KAP levels among caregivers of children with epilepsy in Zibo, China, investigate contributing factors, and analyze the relationships among knowledge, attitude, and practice components. Compared with previous studies in China, such as the work by Cui C et al., which primarily focused on seizure management during acute episodes using a mixed-methods design, the present study extends the scope to a broader spectrum of daily epilepsy management, including long-term caregiving behaviors, social adaptation, and medication-related practices. More importantly, this study introduces structural equation modeling (SEM) to quantitatively examine both the direct and indirect pathways among knowledge, attitude, and practice. By identifying the mediating role of attitude in the knowledge–practice relationship, our findings provide more precise targets for intervention, suggesting that improving caregiving behaviors requires not only enhancing knowledge but also actively addressing parental attitudes and stigma. These findings are anticipated to provide empirical evidence for developing targeted health education programs, strengthening family-centered epilepsy management approaches, and informing communication strategies for clinical healthcare providers.

## Methods

### Study design and participants

This cross-sectional study was conducted between September 2024 and February 2025. We initially performed a random sampling of all three Grade-A hospitals (Zibo First Hospital, Zibo Central Hospital, Zibo Maternity and Child Health Care Hospital) and the specialized epilepsy hospital (Zibo Changguo Hospital) within the Zibo city district. After two hospitals declined to participate due to research schedule conflicts, Zibo First Hospital (a Grade-3 A comprehensive hospital) and Zibo Changguo Hospital (a specialized epilepsy hospital) were confirmed as the study sites. Using convenience sampling, we recruited parents or primary caregivers of children with epilepsy who met the inclusion criteria from the pediatric and neurology outpatient and inpatient departments of these two hospitals. As a hospital-based convenience sample, the study population may not be fully representative of all caregivers of children with epilepsy in China. This study was approved by the Ethics Committee of the Medical Ethics Committee of Zibo First Hospital and informed consent was obtained from all participants via electronic questionnaire prior to data collection [YXLL20240672].

Inclusion criteria required that participants be primary caregivers aged ≥ 18 years of children (aged 0–18 years) diagnosed with epilepsy, including parents or grandparents who held primary responsibility for the child’s daily care and epilepsy management. All participants were capable of understanding the questionnaire and able to complete it independently. To avoid clustering bias, only one primary caregiver per child was enrolled. Informed consent was obtained from all individuals prior to participation. Individuals were excluded from this study based on several criteria. These included: refusal to participate or failure to sign the informed consent form; presence of a co-existing serious chronic disease or developmental disorder in the child; absence of a confirmed epilepsy diagnosis for the child; the respondent not being a legal guardian or primary caregiver; and the presence of cognitive, linguistic, or other barriers that precluded independent completion of the questionnaire. Furthermore, questionnaires with incomplete data on variables essential for the final analysis were also excluded.

### Research instrument

The questionnaire used in this study was developed based on established clinical guidelines and a review of relevant literature. It was subsequently refined through consultation with one pediatric neurology specialist, during which redundant or repetitive items were eliminated, and ambiguous wording was revised to improve clarity and enhance content validity. A pilot test for reliability was conducted with 40 participants. The overall Cronbach’s alpha coefficient for the questionnaire was 0.84, indicating a good level of reliability. The alpha coefficients for the dimensions—knowledge (0.77), attitude (0.72), and practice (0.75)—were all within the acceptable range. These results demonstrate that the questionnaire possesses strong internal consistency and is a reliable and stable instrument suitable for the main study.

The finalized questionnaire comprised four sections: (1) demographic information, including variables such as education level, gender, employment status, type of work unit, and professional title; (2) knowledge; (3) attitude; and (4) practice. The knowledge section assessed two subdomains across 12 items. Each correct response was awarded 2 points, while incorrect or “unclear” responses received 1 point, yielding a possible total score ranging from 12 to 24. This scoring approach was designed to distinguish between complete mastery of correct knowledge and the presence of cognitive gaps or uncertainty, rather than treating all non-correct responses equivalently. The attitude section included 7 items rated on a five-point Likert scale, with responses ranging from “strongly positive” (5 points) to “strongly negative” (1 point), resulting in total scores between 7 and 35. This domain comprised both caregivers’ internal attitudes toward the disease and perceived social stigma, including concerns about how their child may be viewed or treated by others. The practice section also comprised 8 items, of which P8 survey participants or access to knowledge were not scored, and the remaining 7 items similarly assessed using a five-point Likert scale, with response options ranging from “always” (5 points) to “never” (1 point), producing an equivalent score range of 7 to 35. All reverse-scored items were recoded prior to analysis so that higher total practice scores consistently indicate more appropriate and standardized caregiving behaviors. For each domain, a score exceeding 70% of the maximum possible value was defined as indicating adequate knowledge, a positive attitude, or proactive caregiving behavior, respectively, in accordance with commonly adopted thresholds in KAP studies^16^.

### Data collection procedure

Questionnaires were distributed in outpatient clinic settings and were available in both paper-based and electronic formats, depending on participants’ preferences. For those opting for the paper version, research staff provided the questionnaires directly to caregivers who had consented to participate. These were completed independently in designated onsite locations, such as waiting areas or health education rooms. Subsequently, responses were manually entered into a digital database using a double-entry and cross-verification protocol to minimize transcription errors and ensure data accuracy.

The electronic version of the questionnaire was developed using the SoJump platform (SoJump.com; wjx.cn, hereafter referred to as SoJump) and accessed via a Quick Response (QR) code. Participants were instructed to scan the code using their mobile devices, which directed them to the secure online survey interface.

Several measures were implemented to maintain data quality and completeness. Each internet protocol (IP) address was restricted to a single submission to prevent duplicate entries, and all survey items were designated as mandatory to reduce missing data. Upon collection, the research team systematically reviewed all completed questionnaires to verify completeness, assess internal consistency, and confirm logical coherence across responses.

### Sample size calculation

The required sample size for this cross-sectional study was calculated using the standard formula for estimating proportions in finite populations^17^:

$${\text{n }}=\left( {{{\mathrm{Z}}^{\mathrm{2}}} \times {\mathrm{P}} \times \left( {{\text{1 }} - {\text{ P}}} \right)} \right)/{{\mathrm{E}}^{\mathrm{2}}},$$

where Z represents the Z-score corresponding to the desired confidence level (1.96 for 95% confidence), P is the estimated population proportion (set at 0.5 in the absence of prior estimates, as this yields the maximum sample size), and E denotes the acceptable margin of error (commonly set at 0.05). Based on these parameters, the minimum required theoretical sample size was calculated to be 384 participants.

### Statistical methods

Data analysis was performed using STATA version 17.0 (StataCorp, College Station, TX, USA). Continuous variables were expressed as means and standard deviations (mean ± standard deviation, SD), while categorical variables were summarized as frequencies and percentages (n [%]). Normality of continuous variables was assessed using the Shapiro-Wilk test. For group comparisons, analysis of variance (ANOVA) was applied to normally distributed data with homogeneity of variance, whereas the Kruskal–Wallis test was employed for non-normally distributed variables across three or more groups. When comparing two groups, the independent-samples t-test was used for normally distributed variables, and the Wilcoxon rank-sum test (Mann–Whitney U test) was used for non-normally distributed data. Spearman’s rank correlation coefficient was calculated to assess the associations among knowledge, attitude, and practice (KAP) scores. In addition, patients were categorized into monotherapy and polytherapy (≥ 2 drugs) groups based on caregiver-reported medication use, and group differences in KAP scores were compared. Because medication data were caregiver-reported, potential recall bias should be considered when interpreting these findings. Before conducting structural equation modeling, multicollinearity among key variables was assessed using variance inflation factors (VIF) derived from multiple linear regression, and no significant multicollinearity was detected. Structural equation modeling (SEM) was conducted to further explore the directional relationships among the KAP dimensions and to evaluate whether attitude mediates the effect of knowledge on practice. Model fit was assessed using multiple indices, including the root mean square error of approximation (RMSEA), incremental fit index (IFI), Tucker–Lewis index (TLI), and comparative fit index (CFI). A two-sided P-value less than 0.05 was considered to indicate statistical significance, with all P-values reported to three decimal places. Given the exploratory nature of subgroup analyses in this study, no formal adjustment for multiple comparisons was applied. Therefore, the results should be interpreted with caution, particularly for findings with small effect sizes.

## Results

### Demographic information on participants and KAP scores

his study included 385 parents of children with epilepsy, predominantly female (59.5%), married (98.4%), and residing in urban areas (44.7%), and their mean age was 39.72 ± 7.78 years. Most participants held associate/bachelor’s degrees or higher (39.5%) and were employed (49.1%). Their children were mainly school-aged (7–12 years: 45.7%), attended full-time school (73.5%), and experienced seizures > once monthly but < weekly (60.3%) (Table 1). According to report from caregivers, the most common medication used by patients was Levetiracetam (24.68%), followed by Sodium Valproate (22.34%), with all combinations of medications being used in no more than 2% of the cases (Table S1). For further analysis, patients were categorized into monotherapy and polytherapy (≥ 2 drugs) groups based on caregiver-reported medication use, and differences in KAP scores between these groups were examined (Table 2). The mean knowledge, attitudes and practices scores were 19.76 ± 2.68 (possible range: 12–24), 23.43 ± 3.18 (possible range: 7–35), and 23.57 ± 2.52 (possible range: 7–35), respectively.

**Table 1:** Basic information of participants.

*N*=385	*N* (%)
Gender	
Male	156(40.52)
Female	229(59.48)
Age (years), Mean ± SD 39.72 ± 7.78	
Relationship with the patient	
Father	145(37.66)
Mother	215(55.84)
Grandparent	25(6.49)
Residence	
Rural	142(36.88)
Urban	172(44.68)
Suburban	71(18.44)
Education	
Junior high school or below	132(34.29)
Senior high school / Vocational school	101(26.23)
Associate degree / Bachelor’s degree or above	152(39.48)
Current employment status	
Employed	189(49.09)
Self-employed	66(17.14)
Other	130(33.77)
Average monthly income per capita (RMB, ¥)	
< 1500	38(9.87)
1500-4000	132(34.29)
4000-10000	173(44.94)
> 10,000	42(10.91)
Medical expenses over the past year (RMB, ¥)	
< 3000	37(9.61)
3000-5000	111(28.83)
5000-10000	180(46.75)
> 10,000	57(14.81)
Marital status	
Single, divorced, widowed	6(1.56)
Married	379(98.44)
Family history of epilepsy	
Yes	39(10.13)
No	346(89.87)
Patient’s gender	
Male	216(56.1)
Female	169(43.9)
Patient’s age	
Less than 3 years old	37(9.61)
4-6 years old	78(20.26)
7-12 years old	176(45.71)
Over 13 years old	94(24.42)
Patient attending a full-time school	
Yes	283(73.51)
No	102(26.49)
Frequency of epileptic seizures	
More than once per day	18(4.68)
More than once per week	57(14.81)
More than once per month but less than once per week	232(60.26)
Less than once per month	24(6.23)
Other	54(14.03)
Birth history	
Full-term	361(93.77)
Premature	24(6.23)
Perinatal brain injury	
Yes	13(3.38)
No	372(96.62)
The frequency of seizures significantly decreased after taking medication	
Yes	343(89.09)
No	42(10.91)

Other includes heterogeneous clinical patterns that do not fit standard frequency categories, including: (1) prolonged remission (e.g., seizure-free for ≥1–2 years but still under treatment or follow-up); (2) seizure clusters or highly irregular patterns (e.g., long seizure-free intervals interrupted by multiple seizures within a short period); and (3) mixed seizure types or frequency patterns (e.g., different seizure types occurring at different frequencies or seizures triggered under specific conditions). Seizure frequency reduction after medication was self-reported by caregivers based on their daily observations and was not independently verified using clinical records.

**Table 2:** Participants’ KAP scores.

*N* = 385	Knowledge score		Attitude score		Practice score	
	Mean ± SD	*P*	Mean ± SD	*P*	Mean ± SD	*P*
Total score	19.76 ± 2.68		23.43 ± 3.18		23.57 ± 2.52	
Gender		0.278		0.914		0.452
Male	19.96 ± 2.54		23.46 ± 2.88		23.63 ± 2.16	
Female	19.62 ± 2.76		23.41 ± 3.36		23.52 ± 2.74	
Relationship with the patient		0.113		0.893		0.723
Father	20.04 ± 2.60		23.33 ± 2.86		23.57 ± 2.10	
Mother	19.62 ± 2.74		23.50 ± 3.20		23.52 ± 2.75	
Grandparent	19.24 ± 2.50		23.36 ± 4.54		23.88 ± 2.77	
Residence		0.001		0.016		0.708
Rural	19.26 ± 2.73		23.33 ± 2.95		23.47 ± 2.85	
Urban	20.25 ± 2.68		23.83 ± 3.37		23.59 ± 2.28	
Suburban	19.57 ± 2.38		22.66 ± 3.00		23.67 ± 2.38	
Education		0.036		0.003		0.159
Junior high school or below	19.40 ± 2.55		23 ± 3.14		23.44 ± 2.40	
Senior high school / Vocational school	19.72 ± 2.91		23.02 ± 3.22		23.06 ± 2.70	
Associate degree / Bachelor’s degree or above	20.09 ± 2.60		24.07 ± 3.08		24.00 ± 2.44	
Current employment status		<0.001		<0.001		0.029
Employed	20.32 ± 2.58		24.02 ± 2.99		23.97 ± 2.28	
Self-employed	19.69 ± 2.42		23.77 ± 2.73		23.06 ± 1.76	
Other	18.97 ± 2.76		22.40 ± 3.41		23.23 ± 3.05	
Average monthly income per capita		0.117		0.260		0.036
< 1500	18.86 ± 2.78		23.65 ± 3.45		24.36 ± 3.01	
1500-4000	19.96 ± 2.40		23.31 ± 2.97		23.85 ± 2.52	
4000-10000	19.76 ± 2.72		23.29 ± 3.24		23.27 ± 2.43	
> 10,000	19.90 ± 3.17		24.16 ± 3.26		23.16 ± 2.20	
Medical expenses over the past year		0.207		0.252		0.537
< 3000	20.08 ± 2.38		23.54 ± 2.26		23.62 ± 2.36	
3000-5000	19.63 ± 2.71		23.75 ± 3.03		23.51 ± 2.55	
5000-10000	19.92 ± 2.75		23.52 ± 3.20		23.47 ± 2.54	
> 10,000	19.26 ± 2.56		22.43 ± 3.71		23.94 ± 2.52	
Marital status		0.988		0.193		0.049
Single, divorced, widowed	20 ± 2.09		25.16 ± 4.70		24.5 ± 0.83	
Married	19.75 ± 2.69		23.40 ± 3.14		23.55 ± 2.53	
Family history of epilepsy		0.573		0.846		0.977
Yes	20.25 ± 1.94		23.66 ± 3.87		23.94 ± 2.45	
No	19.70 ± 2.75		23.40 ± 3.09		23.52 ± 2.53	
Patient information						
Patient’s gender		0.379		0.122		0.869
Male	19.80 ± 2.79		23.63 ± 3.14		23.54 ± 2.56	
Female	19.70 ± 2.54		23.17 ± 3.21		23.59 ± 2.48	
Patient’s age		<0.001		0.174		<0.001
Less than 3 years old	19.08 ± 2.46		22.51 ± 3.99		24.27 ± 2.65	
4-6 years old	18.87 ± 2.83		23.01 ± 3.37		22.73 ± 2.78	
7-12 years old	19.91 ± 2.54		23.77 ± 2.95		23.92 ± 2.47	
Over 13 years old	20.47 ± 2.67		23.51 ± 2.99		23.32 ± 2.12	
Patient attending a full-time school		< 0.001		0.002		0.277
Yes	20.12 ± 2.65		23.76 ± 2.89		23.55 ± 2.42	
No	18.76 ± 2.51		22.50 ± 3.71		23.61 ± 2.78	
Frequency of epileptic seizures		0.153		< 0.001		< 0.001
More than once per day	18.88 ± 1.77		21.16 ± 2.99		24.72 ± 3.59	
More than once per week	20 ± 2.30		23.31 ± 2.75		23.78 ± 1.85	
More than once per month but less than once per week	19.71 ± 2.97		23.18 ± 2.87		22.81 ± 2.02	
Less than once per month	20.41 ± 2.10		25.58 ± 4.32		27.25 ± 3.23	
Other	19.70 ± 2.10		24.42 ± 3.60		24.55 ± 2.40	
Birth history		0.028		0.526		0.049
Full-term	19.68 ± 2.70		23.44 ± 3.24		23.61 ± 2.58	
Premature	20.91 ± 2.01		23.20 ± 1.93		22.91 ± 1.13	
Perinatal brain injury		0.214		0.971		0.256
Yes	20.30 ± 3.32		23.30 ± 4.49		24.69 ± 3.06	
No	19.74 ± 2.66		23.43 ± 3.13		23.52 ± 2.49	
The frequency of seizures significantly decreased after taking medication		0.001		0.969		0.772
Yes	19.90 ± 2.66		23.38 ± 3.08		23.51 ± 2.39	
No	18.57 ± 2.52		23.83 ± 3.88		23.97 ± 3.41	
What medications are taken orally		< 0.001		0.887		< 0.001
Monotherapy	19.83 ± 2.42		23.43 ± 3.22		23.95 ± 2.50	
Multidrug combination therapy	19.99 ± 3.40		23.37 ± 3.14		22.50 ± 2.36	
Others	17.20 ± 1.86		23.80 ± 2.73		22.00 ± 1.41	

The maximum possible score ranges were as follows: knowledge (12–24), attitude (7–35), and practice (7–35). All reverse-scored items were recoded before total score calculation; therefore, higher practice scores uniformly indicate better caregiving practices.

### Knowledge scores

Knowledge scores differed significantly by residence (urban vs. rural), education level (associate/bachelor’s degree or higher vs. junior high or below), employment status (employed vs. other), patient’s age (over 13 years vs. 4–6 years), school attendance (yes vs. no), birth history (premature vs. full-term), and medication efficacy (seizure reduction vs. no reduction).

### Attitude scores

Attitude scores varied significantly by residence (urban vs. suburban), education (associate/bachelor’s degree or higher vs. senior high), employment (employed vs. other), seizure frequency (less than monthly vs. more than once daily), and school attendance (yes vs. no).

### Practice scores

Practice scores showed significant associations with employment status (employed vs. self-employed), income (<¥1500 vs. >¥10,000), marital status (single/divorced vs. married), patient’s age (< 3 years vs. 4–6 years), seizure frequency (less than monthly vs. more than once monthly but less than weekly), and birth history (full-term vs. premature) (Table 2).

It is important to note that the sample size of certain subgroups, such as the single/divorced group (*n* = 6) and the perinatal brain injury group (*n* = 13), was very small; therefore, the corresponding statistical comparisons should be interpreted with caution due to potential instability. Although some differences reached statistical significance, the corresponding effect sizes were generally small, and the absolute differences in KAP scores were minimal (often less than 1 point), indicating limited practical significance.

### Knowledge, attitude, and practice

**Fig. 1:**
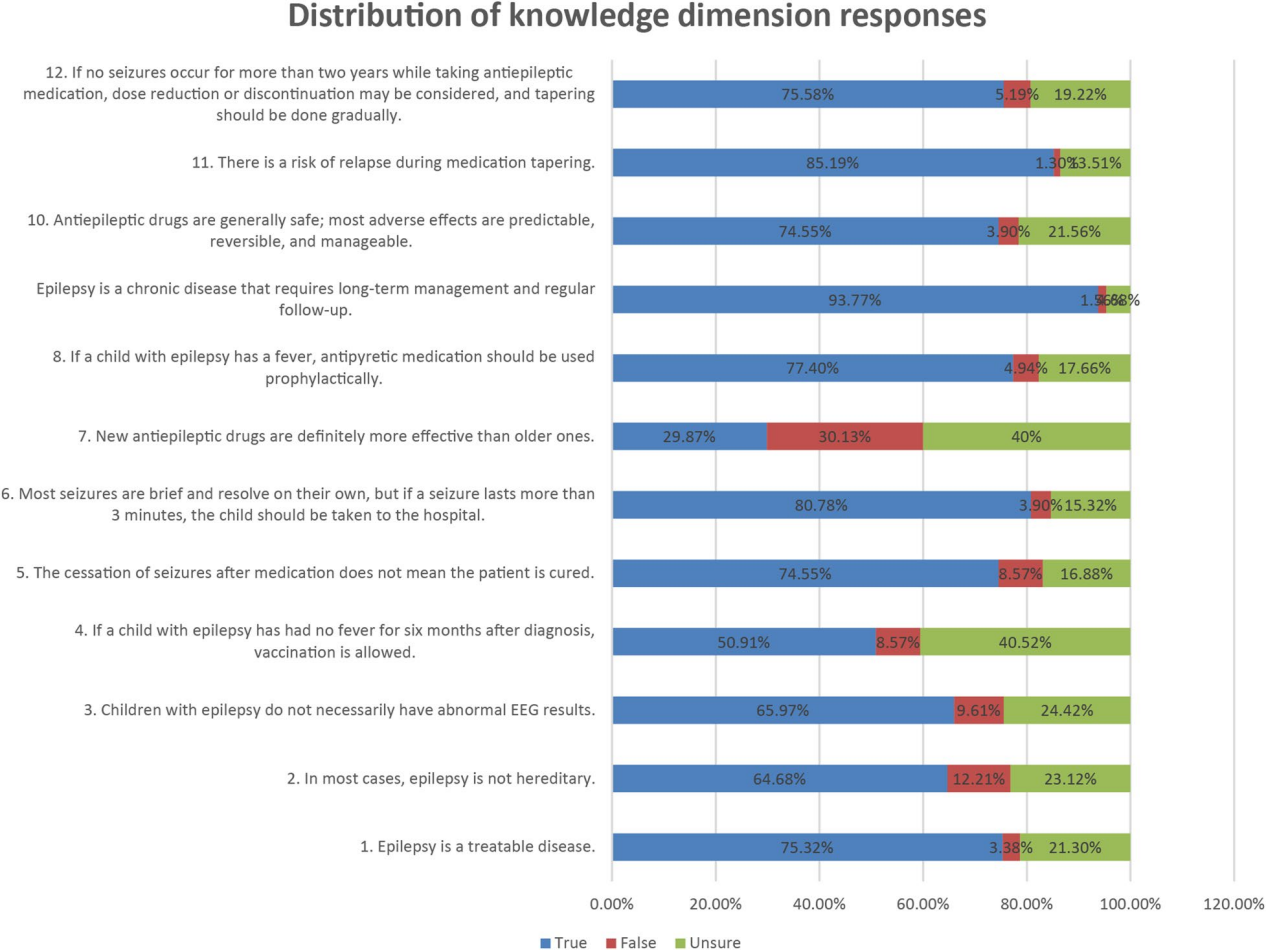
Distribution of knowledge dimension responses.

The distribution of knowledge dimensions showed that the three questions with the highest number of participants choosing the ‘Unsure’ option were ‘If a child with epilepsy has had no fever for six months after diagnosis, vaccination is allowed.’ (K4) with 40.52%, ‘New antiepileptic drugs are definitely more effective than older ones.’ (K7) with 40%, and ‘Children with epilepsy do not necessarily have abnormal EEG results.’ (K3) with 24.42% (Fig. 1).

**Fig. 2:**
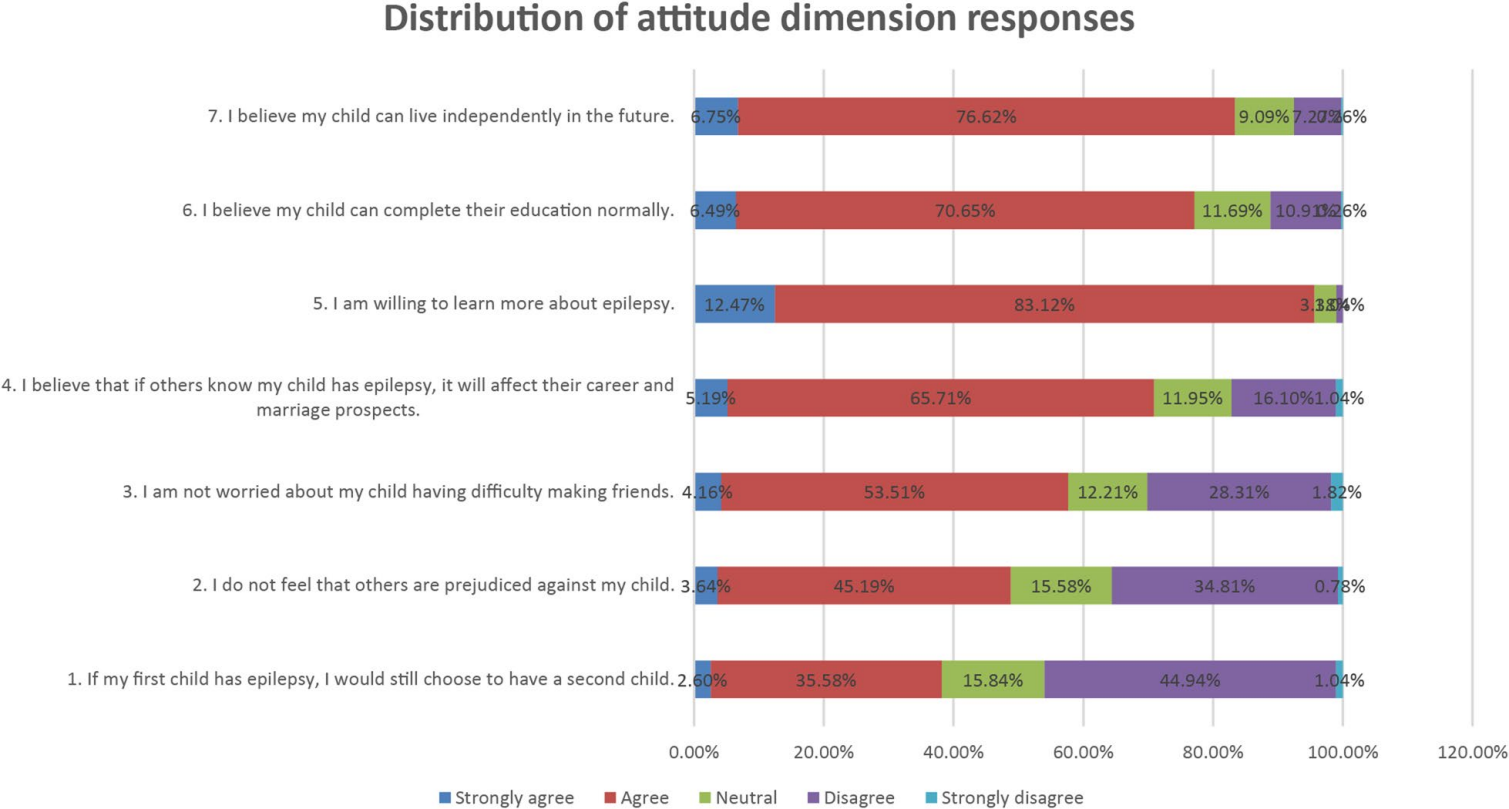
Distribution of attitude dimension responses.

When it comes to related attitudes, 70.9% believed that epilepsy would affect their child’s career and marriage prospects (A4), 35.59% thought their children would be prejudiced against by others (A2), and 30.13% were concerned that their children would have difficulties making friends (A3) (Fig. 2).

**Fig. 3:**
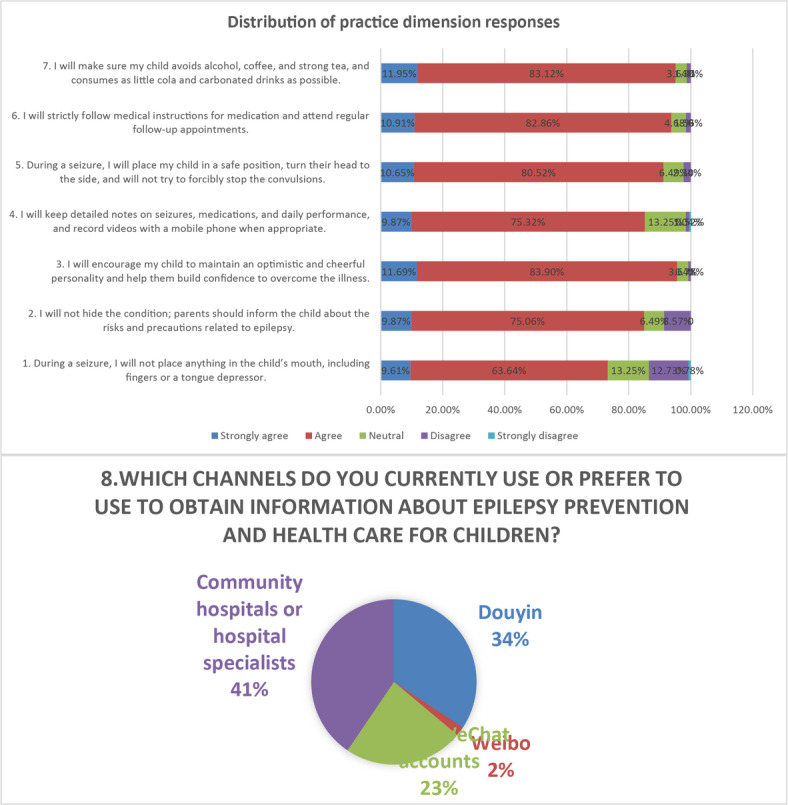
Distribution of practice dimension responses.

Responses on the practice dimension showed that 13.53% and 8.57% chose ‘disagree’ or ‘strongly disagree’ for P1 and P2, respectively, indicating that they still put things in their children’s mouths during seizures and hide their condition. In addition, 13.25% were neutral and 1.94% were opposed regarding take detailed notes on seizures, medications, and daily performance (P4) (Fig. 3).

### Correlation analysis

Further correlation analysis revealed statistically significant but weak to moderate positive correlations between knowledge and attitude (*r* = 0.277, 95% CI: 0.1820–0.3667, *P* < 0.001), as well as between knowledge and practice (*r* = 0.260, 95% CI: 0.1642–0.3507, *P* < 0.001). Additionally, attitude was weakly positively correlated with practice (*r* = 0.171, 95% CI: 0.0721–0.2662, *P* < 0.001) (Table 3).

**Table 3:** Correlation analysis of KAP scores.

	Knowledge	Attitude	Practice
Knowledge	1		
Attitude	0.2769 (0.1820-0.3667)	1	
Practice	0.2599 (0.1642-0.3507)	0.1708 (0.0721-0.2662)	1

Values are presented as correlation coefficients (r) with 95% confidence intervals.

### CFA and SEM analysis

**Fig. 4:**
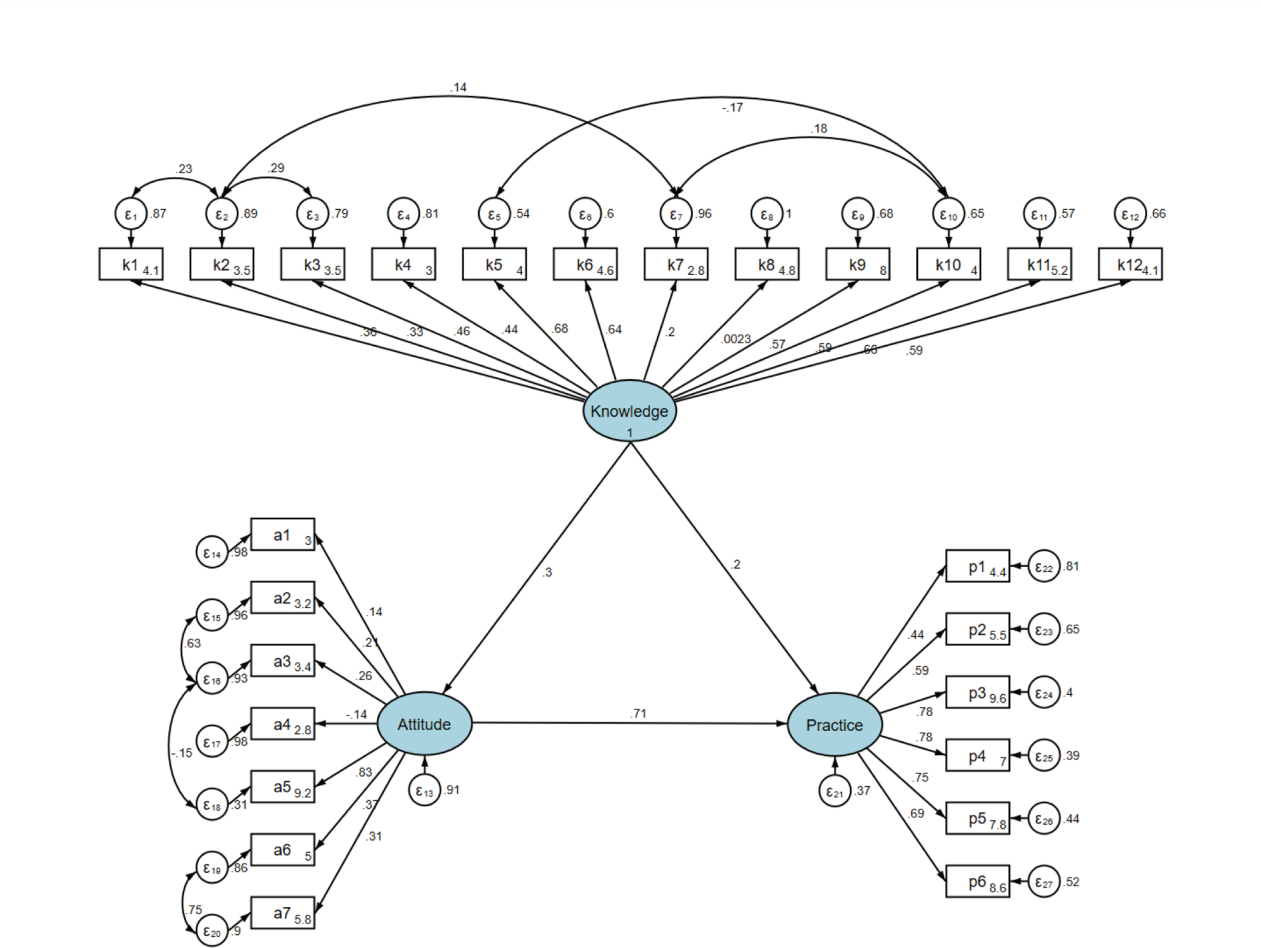
SEM path.

Prior to structural equation modeling, confirmatory factor analysis (CFA) was conducted to evaluate the measurement model. The results showed that most items had statistically significant factor loadings, and the overall model demonstrated an acceptable fit (χ^2^/df = 2.589, RMSEA = 0.064, CFI = 0.853, TLI = 0.833, SRMR = 0.082). Detailed factor loadings and construct validity indices are presented in Tables S2–S4. The structural model was then constructed to examine the relationships among knowledge, attitude, and practice. Standardized path coefficients indicated that knowledge had a significant direct effect on attitude (β = 0.303, *P* < 0.001) and practice (β = 0.204, *P* = 0.001), while attitude had a strong direct effect on practice (β = 0.708, *P* < 0.001). In addition, knowledge showed a significant indirect effect on practice through attitude (β = 0.215, *P* < 0.001), indicating a mediating role of attitude (Table 4 and Fig. 4). Because composite scores were used to construct the structural model, the model was just-identified (df = 0); therefore, global fit indices were not considered informative.

**Table 4:** SEM results.

Model paths		Total effects		Direct Effect		Indirect effect	
		β (95%CI)	*P*	β (95%CI)	*P*	β (95%CI)	*P*
Attitude	Knowledge	0.303 (0.165, 0.442)	< 0.001	0.303 (0.165, 0.442)	< 0.001		
Practice	Knowledge	0.418 (0.315, 0.521)	< 0.001	0.204 (0.080, 0.327)	0.001	0.215 (0.102, 0.328)	< 0.001
	Attitude	0.708 (0.585, 0.831)	< 0.001	0.708 (0.585, 0.831)	< 0.001		

## Discussion

Although caregivers demonstrated knowledge levels that met the threshold for adequacy, important knowledge gaps persisted, particularly in key areas of disease management, while negative attitudes and suboptimal caregiving practices remained prevalent. These findings highlight the need for targeted educational interventions that not only improve factual knowledge but also address stigma-related attitudes and promote effective epilepsy management behaviors in daily caregiving.

The results of this study are particularly prominent in the context of the long-term management of epilepsy in children, as childhood is a critical stage in dynamic development. Unlike adult epilepsy, the causes of childhood epilepsy, the types of epileptic syndromes, and their long-term effects on cognition and behavior are more complex and heterogeneous. The pattern revealed in this study—where parents have basic knowledge but still hold negative attitudes and adopt inappropriate practices—reflects a core challenge in the care of chronic childhood diseases: information perception is often disconnected from emotional, cultural, and behavioral barriers. The present findings reveal a pattern commonly reported in family-based epilepsy management studies: caregivers tend to possess a general understanding of the disorder, yet continue to express negative attitudes and adopt only partially appropriate care strategies. This mismatch has been widely noted in studies of chronic childhood conditions, where informational awareness is often outpaced by emotional, cultural, or behavioral constraints^18,19^. Although most caregivers in this sample correctly identified epilepsy as a chronic yet manageable illness, their responses on attitudinal and behavioral items suggest persistent concerns about social stigma, long-term dependency, and perceived unpredictability. Related research suggests that in contexts where epilepsy remains socially sensitive, caregivers may hesitate to engage fully in disclosure or proactive behavior, regardless of clinical knowledge^20,21^.

Model-based results reinforce this interpretation. Correlation analysis showed statistically significant but weak positive associations among the KAP dimensions, with the strongest—yet still modest—association observed between knowledge and attitude. Structural equation modeling further demonstrated that knowledge had a direct effect on both attitude and practice, while attitude also exerted a strong influence on practice. Although the indirect effect of knowledge on practice through attitude was statistically significant, its magnitude should be interpreted with caution, and emphasis should be placed on the relative effect sizes rather than statistical significance alone. This finding suggests only partial and modest mediation, rather than a strong restructuring of classical KAP theory, indicating that the pathway from knowledge to practice is more complex than a simple linear progression. Instead, the present data support a more complex framework, one in which knowledge may prompt behavioral adherence independently of emotional acceptance, especially in situations where social pressure or chronic stress dilute the motivational role of attitude^22,23^. Research in epilepsy and other stigmatized conditions has similarly questioned whether attitude consistently mediates the knowledge-behavior link, particularly among caregivers navigating long-term uncertainty and isolation^24,25^.

When stratified by caregiver and child characteristics, the data point to several structural differences in KAP performance. Urban residence, higher education, and full-time employment were each associated with better outcomes across knowledge and attitude scores. These findings are consistent with prior evidence that caregiver literacy and social capital enhance both health information uptake and the internalization of medical norms^26^. In contrast, income did not show significant associations with knowledge or attitude scores, but it was significantly associated with practice scores. This absence of effect highlights a recurrent theme in epilepsy care literature: financial status may influence access to services but does not, in itself, resolve the cultural and emotional barriers that shape how families perceive and manage epilepsy^27,28^. Interestingly, caregivers of children with more frequent seizures demonstrated better practice scores. This counterintuitive finding may be explained by increased healthcare exposure, as these caregivers are more likely to seek medical attention, encounter emergency situations, and receive repeated guidance from healthcare professionals. Over time, such repeated exposure may enhance their practical caregiving skills and confidence. Furthermore, several statistically significant subgroup differences corresponded to very small absolute score variations, suggesting that these findings should be interpreted cautiously and not overemphasized in terms of practical importance.

Medication patterns, as reported in Table S1, were included primarily to describe the clinical background of the study population rather than to evaluate physicians’ prescribing behaviors. Given that these data were caregiver-reported, they may be subject to recall bias and should be interpreted with caution. In this study, treatment complexity (monotherapy vs. polytherapy) was considered as a potential contextual factor and was further analyzed in relation to KAP scores, with the aim of exploring whether more complex treatment regimens are associated with differences in caregiver knowledge, attitudes, or practices.

Attitudinal data suggest a somewhat conflicted emotional stance toward epilepsy. While caregivers overwhelmingly expressed a desire to learn more about the condition, large numbers endorsed concerns about social judgment, future marriage prospects, and peer interaction. Notably, the high proportion of caregivers expressing concerns about marriage and career prospects reflects the persistence of epilepsy-related stigma, which may have long-term implications for both caregiver psychological burden and children’s social integration. These fears reflect broader cultural narratives around neurological illness as a source of shame or social disruption, particularly in settings where mental and neurological conditions remain poorly integrated into public health discourse^29^. Importantly, some of these responses reflect caregivers’ perceptions of societal attitudes (perceived stigma) rather than purely their own internal beliefs, indicating that external social expectations may play a significant role in shaping caregiver psychology and behavior. Even as some respondents affirmed belief in their child’s educational and personal potential, this did not eliminate their reservations about openness or reproductive decision-making. In related studies, such contradictory beliefs have been interpreted as markers of emotional coping in the absence of consistent institutional or psychosocial support^30^. For parents of children with epilepsy, these deep concerns about the future are a direct reflection of the core challenges of long-term management – not only dealing with the disease at hand, but also planning for their child’s social integration and quality of life for decades to come.

Behavioral practices, as recorded in the supplementary practice table, showed relatively high adherence in certain areas—especially with respect to medication and seizure response positioning. However, a substantial portion of caregivers continued to endorse unsafe or outdated practices, such as inserting objects into the mouth during seizures. This particular misconception has proven durable in multiple studies, often attributed to long-standing folk beliefs and insufficient reinforcement of clinical protocols during routine visits^31,32^. While knowledge of correct actions may be present, high-stress situations such as active seizures demand practiced confidence, not just cognitive awareness. This gap between declarative knowledge and automatic behavior suggests the need for repeated training and scenario-based education. This finding underscores a critical gap between knowledge and appropriate behavioral execution, emphasizing the need for repeated, practice-oriented training rather than knowledge dissemination alone.

These findings suggest several practical steps. First, caregiver education must extend beyond seizure control to include structured guidance on medication decisions, tapering processes, and long-term developmental planning. One-off explanations during diagnosis or follow-up are unlikely to address the variety of questions and challenges that emerge over time. Repetition, interactive demonstrations, and low-literacy visual aids should be incorporated into caregiver training, especially for those with limited formal education or high emotional stress. Where possible, peer-led models—featuring caregivers with lived experience—may offer additional credibility and relevance^33,34^. In addition, structured discharge education programs, hospital-based counseling sessions, and community or school-based support groups may provide practical and sustainable platforms for improving caregiver knowledge, attitudes, and behaviors.

Second, clinical services should incorporate structured counseling on stigma, disclosure, and family decision-making. Many caregivers expressed conflicting feelings about whether to disclose the diagnosis or expand their family. These are not merely emotional matters but deeply practical ones, affecting treatment continuity, school integration, and long-term support. Clinics should offer referral pathways to social work or psychology services and should proactively initiate discussions about stigma and social participation, rather than waiting for caregivers to raise such concerns^35,36^.

Third, variation in medication use patterns suggests the need for tighter feedback loops between physicians and families. When caregivers begin modifying treatment on their own or incorporating unverified therapies, this often reflects unmet needs for reassurance and understanding. Scheduled check-ins, home-based follow-up, and shared decision-making tools may help address these uncertainties before they lead to non-adherence or potentially harmful substitutions^32,37^.

This study has several limitations that should be considered when interpreting the findings. First, the use of hospital-based convenience sampling from only two centers may limit external validity and introduce potential selection bias. In addition, the sample was predominantly composed of married caregivers and mothers, reflecting typical family structures and caregiving roles in the study setting; however, the findings may therefore be more generalizable to two-parent families, while single-parent caregivers, who may face different psychological and socioeconomic challenges, were underrepresented. Second, as a cross-sectional survey, the study captures parental KAP at a single point in time and cannot establish causality or temporal changes. Third, the use of self-reported questionnaires may introduce response bias, as participants may overestimate socially desirable behaviors or underreport negative attitudes. Furthermore, because the data were based on caregiver reports, detailed clinical classifications (e.g., seizure type or epilepsy syndrome) could not be reliably obtained; therefore, seizure frequency and treatment complexity were used as proxy indicators of disease severity. Fourth, multiple subgroup comparisons were conducted without formal adjustment for multiple testing, which may increase the risk of type I error. In addition, some subgroup analyses were based on relatively small sample sizes, which may further limit the stability and reliability of the corresponding estimates. Therefore, statistically significant findings, particularly those with small effect sizes, should be interpreted with caution. Finally, the study was conducted in two hospitals within a single region, which may limit the generalizability of the findings to other geographic or socioeconomic populations.

In conclusion, parents of children with epilepsy demonstrated generally adequate levels of knowledge but held predominantly negative attitudes and engaged in suboptimal caregiving practices. These findings highlight the need for integrated educational interventions that not only improve knowledge but also address attitudes, although the mediating role of attitude appears to be modest, suggesting that improving caregiving behavior may require addressing multiple factors beyond knowledge alone.

## Supplementary Information

Below is the link to the electronic supplementary material.

Supplementary Material 1

## Data Availability

All data generated or analysed during this study are included in this published article.
