# Supplementary material for: Knowledge, attitudes, and practices regarding childhood epilepsy among parents of children with epilepsy: a questionnaire-based study
Source: Sci Rep. 2026 May 18;16:22600. doi: 10.1038/s41598-026-50907-2 (PMC13381562; doi:10.1038/s41598-026-50907-2)
Supplement: Supplementary file 1 — Supplementary Material 1 [file 41598_2026_50907_MOESM1_ESM.docx]

**Supplementary table 1. Caregivers’ self-reported medications used by patients**

Therapy category summary: Monotherapy: n = 274 (71.17%); Polytherapy (≥2 drugs): n = 103 (26.75%), Dual therapy: n = 77 (20.00%), and Triple therapy: n = 26 (6.75%); Unspecified/No medication: n = 8 (2.08%)

| Therapy Category | Standardized Medication Name | N | Percent (%) |
| --- | --- | --- | --- |
| Monotherapy |  |  |  |
|  | Levetiracetam | 95 | 24.68 |
|  | Sodium Valproate | 86 | 22.34 |
|  | Oxcarbazepine | 32 | 8.31 |
|  | Lamotrigine | 23 | 5.97 |
|  | Carbamazepine | 11 | 2.86 |
|  | Lacosamide | 11 | 2.86 |
|  | Zonisamide | 7 | 1.82 |
|  | Topiramate | 4 | 1.04 |
|  | Perampanel | 4 | 1.04 |
|  | Clobazam | 1 | 0.26 |
| Dual Therapy |  |  |  |
|  | Sodium Valproate + Levetiracetam | 7 | 1.82 |
|  | Sodium Valproate + Lamotrigine | 7 | 1.82 |
|  | Oxcarbazepine + Sodium Valproate | 7 | 1.82 |
|  | Oxcarbazepine + Levetiracetam | 6 | 1.56 |
|  | Sodium Valproate + Topiramate | 6 | 1.56 |
|  | Sodium Valproate + Lacosamide | 5 | 1.3 |
|  | Levetiracetam + Lacosamide | 4 | 1.04 |
|  | Levetiracetam + Topiramate | 2 | 0.52 |
|  | Zonisamide + Sodium Valproate | 2 | 0.52 |
|  | Sodium Valproate + Perampanel | 2 | 0.52 |
|  | Sodium Valproate + Clonazepam | 2 | 0.52 |
|  | Oxcarbazepine + Topiramate | 2 | 0.52 |
|  | Levetiracetam + Oxcarbazepine | 2 | 0.52 |
|  | Carbamazepine + Zonisamide | 1 | 0.26 |
|  | Carbamazepine + Lamotrigine | 1 | 0.26 |
|  | Vigabatrin + Sodium Valproate | 1 | 0.26 |
|  | Perampanel + Sodium Valproate | 1 | 0.26 |
|  | Oxcarbazepine + Lacosamide | 1 | 0.26 |
|  | Oxcarbazepine + Carbamazepine | 1 | 0.26 |
|  | Oxcarbazepine + Perampanel | 1 | 0.26 |
|  | Levetiracetam + Clonazepam | 1 | 0.26 |
|  | Levetiracetam + Clobazam | 1 | 0.26 |
|  | Phenobarbital + Topiramate | 1 | 0.26 |
| Triple Therapy |  |  |  |
|  | Sodium Valproate + Perampanel + Levetiracetam | 3 | 0.78 |
|  | Sodium Valproate + Levetiracetam + Topiramate | 3 | 0.78 |
|  | Sodium Valproate + Lamotrigine + Levetiracetam | 2 | 0.52 |
|  | Sodium Valproate + Lamotrigine + Clobazam | 1 | 0.26 |
|  | Oxcarbazepine + Sodium Valproate + Perampanel | 1 | 0.26 |
|  | Topiramate + Perampanel + Sodium Valproate | 1 | 0.26 |
|  | Levetiracetam + Lamotrigine + Perampanel | 1 | 0.26 |
|  | Levetiracetam + Sodium Valproate + Oxcarbazepine | 1 | 0.26 |
|  | Levetiracetam + Vigabatrin + Perampanel | 1 | 0.26 |
|  | Sodium Valproate + Lamotrigine + Topiramate | 1 | 0.26 |
|  | Sodium Valproate + Vigabatrin + Topiramate | 1 | 0.26 |
|  | Sodium Valproate + Topiramate + Clobazam | 1 | 0.26 |
|  | Lamotrigine + Clobazam + Levetiracetam | 1 | 0.26 |
| Regimens Containing Non-Antiepileptic Drugs/Therapies |  |  |  |
|  | Levetiracetam + Traditional Chinese Medicine | 1 | 0.26 |
|  | Sodium Valproate + Yinaoling (a traditional remedy) | 2 | 0.52 |
|  | Levetiracetam + Perampanel + Topiramate + Ketogenic Diet | 1 | 0.26 |
|  | Levetiracetam + Sodium Valproate + Vigabatrin | 1 | 0.26 |
| Unspecified/No Medication |  |  |  |
|  | None | 7 | 1.82 |
|  | Triple therapy (medications not specified) | 1 | 0.26 |
|  | Prescription drugs (unspecified) | 1 | 0.26 |
|  | Not filled in | 1 | 0.26 |
| Total |  | 385 | 100 |

_Note: “Unspecified/No medication” includes patients with missing medication information, those not receiving regular treatment, or those unable to recall medication details._

**Supplementary Table S2. Standardized and Unstandardized Factor Loadings for the Measurement Model**

| **Latent Construct** | **Item** | **Estimate** | **SE** | ***Z*-value** | ***P*-value** | **Standardized Loading (*λ*)** |
| --- | --- | --- | --- | --- | --- | --- |
| **Knowledge** | K1 | 0.169 | 0.023 | 7.303 | < 0.001 | 0.393 |
|  | K2 | 0.190 | 0.026 | 7.386 | < 0.001 | 0.397 |
|  | K3 | 0.236 | 0.025 | 9.506 | < 0.001 | 0.497 |
|  | K4 | 0.228 | 0.026 | 8.618 | < 0.001 | 0.456 |
|  | K5 | 0.280 | 0.022 | 12.943 | < 0.001 | 0.644 |
|  | K6 | 0.245 | 0.020 | 12.397 | < 0.001 | 0.622 |
|  | K7 | 0.114 | 0.025 | 4.511 | < 0.001 | 0.249 |
|  | K8 | 0.003 | 0.012 | 0.271 | 0.786 | 0.015 |
|  | K9 | 0.133 | 0.012 | 10.713 | < 0.001 | 0.551 |
|  | K10 | 0.255 | 0.022 | 11.524 | < 0.001 | 0.586 |
|  | K11 | 0.224 | 0.018 | 12.623 | < 0.001 | 0.631 |
|  | K12 | 0.258 | 0.022 | 11.886 | < 0.001 | 0.601 |
| **Attitude** | A1 | 0.069 | 0.053 | 1.323 | 0.186 | 0.071 |
|  | A2 | 0.443 | 0.050 | 8.889 | < 0.001 | 0.454 |
|  | A3 | 0.576 | 0.048 | 11.938 | < 0.001 | 0.586 |
|  | A4 | -0.017 | 0.046 | -0.369 | 0.712 | -0.020 |
|  | A5 | 0.125 | 0.023 | 5.367 | < 0.001 | 0.284 |
|  | A6 | 0.683 | 0.033 | 20.819 | < 0.001 | 0.910 |
|  | A7 | 0.554 | 0.030 | 18.510 | < 0.001 | 0.834 |
| **Practice** | P1 | 0.358 | 0.044 | 8.143 | < 0.001 | 0.425 |
|  | P2 | 0.407 | 0.035 | 11.736 | < 0.001 | 0.583 |
|  | P3 | 0.320 | 0.020 | 16.354 | < 0.001 | 0.754 |
|  | P4 | 0.446 | 0.025 | 17.598 | < 0.001 | 0.795 |
|  | P5 | 0.388 | 0.024 | 16.370 | < 0.001 | 0.755 |
|  | P6 | 0.327 | 0.022 | 14.822 | < 0.001 | 0.701 |

*Note: SE = Standard Error;λ corresponds to the standardized factor loadings (Std.all).*

**Supplementary Table S3. Measurement Model Fit Indices**

| **Fit Index** | **Observed Value** |
| --- | --- |
| χ2 | 683.608 |
| Degrees of freedom (*df*) | 264 |
| χ2/*df* | 2.589 |
| *P*-value | < 0.001 |
| RMSEA | 0.064 |
| SRMR | 0.082 |
| CFI | 0.853 |
| TLI | 0.833 |

*Note: RMSEA = Root Mean Square Error of Approximation; SRMR = Standardized Root Mean Square Residual; CFI = Comparative Fit Index; TLI = Tucker-Lewis Index.*

**Supplementary Table S4. Construct Reliability and Average Variance Extracted (AVE)**

| **Latent Construct** | **Cronbach's**  **α*α*** | **Composite Reliability (CR)** | **Average Variance Extracted (AVE)** |
| --- | --- | --- | --- |
| Knowledge | 0.780 | 0.740 | 0.247 |
| Attitude | 0.611 | 0.226 | 0.083 |
| Practice | 0.790 | 0.794 | 0.394 |

*Note: CR (Composite Reliability) assesses the internal consistency of the indicators measuring a given factor; AVE (Average Variance Extracted) measures the amount of variance captured by a construct in relation to the amount of variance due to measurement error. The relatively low AVE and CR values for certain constructs may reflect the exploratory nature of KAP measurements and the heterogeneity of questionnaire items.*
